# Measles Case Fatality Rate in Bihar, India, 2011–12

**DOI:** 10.1371/journal.pone.0096668

**Published:** 2014-05-13

**Authors:** Manoj V. Murhekar, Mohammad Ahmad, Hemant Shukla, Kunwar Abhishek, Robert T. Perry, Anindya S. Bose, Rahul Shimpi, Arun Kumar, Kanagasabai Kaliaperumal, Raman Sethi, Vadivoo Selvaraj, Pattabi Kamaraj, Satyabrata Routray, Vidya Nand Das, Nata Menabde, Sunil Bahl

**Affiliations:** 1 National Institute of Epidemiology, ICMR, Chennai, India; 2 World Health Organization- Country Office for India, National Polio Surveillance Project, New Delhi, India; 3 World Health Organization, Geneva, Switzerland; 4 Bill and Melinda Gates Foundation, New Delhi, India; 5 Rajendra Memorial Research Institute for Medical Sciences, Patna, Bihar; University of Massachusetts, United States of America

## Abstract

**Background:**

Updated estimates of measles case fatality rates (CFR) are critical for monitoring progress towards measles elimination goals. India accounted for 36% of total measles deaths occurred globally in 2011. We conducted a retrospective cohort study to estimate measles CFR and identify the risk factors for measles death in Bihar–one of the north Indian states historically known for its low vaccination coverage.

**Methods:**

We systematically selected 16 of the 31 laboratory-confirmed measles outbreaks occurring in Bihar during 1 October 2011 to 30 April 2012. All households of the villages/urban localities affected by these outbreaks were visited to identify measles cases and deaths. We calculated CFR and used multivariate analysis to identify risk factors for measles death.

**Results:**

The survey found 3670 measles cases and 28 deaths (CFR: 0.78, 95% confidence interval: 0.47–1.30). CFR was higher among under-five children (1.22%) and children belonging to scheduled castes/tribes (SC/ST, 1.72%). On multivariate analysis, independent risk factors associated with measles death were age <5 years, SC/ST status and non-administration of vitamin A during illness. Outbreaks with longer interval between the occurrence of first case and notification of the outbreak also had a higher rate of deaths.

**Conclusions:**

Measles CFR in Bihar was low. To further reduce case fatality, health authorities need to ensure that SC/ST are targeted by the immunization programme and that outbreak investigations target for vitamin A treatment of cases in high risk groups such as SC/ST and young children and ensure regular visits by health-workers in affected villages to administer vitamin A to new cases.

## Introduction

Estimates of measles-related deaths are critical for monitoring progress towards global measles elimination goals [1], [2]. However, absence of complete and reliable mortality data, particularly from countries with high disease burden, has been a major challenge in measuring the measles mortality reduction goals. In many developing countries, deaths from measles are not routinely reported and measles cases are substantially under-reported. Measles case fatality rates (CFRs) are a key input in models used by the World Health Organization (WHO) to estimate measles related deaths [1], [3]. Hence, generating reliable estimates of measles CFR in a variety of settings and at different periods of time at the national and sub-national levels is important for monitoring the impact of national immunization programmes.

India has had low coverage with measles-containing vaccine (MCV), 74% among children aged between 12–23 months in 2008 [4], and was one of the last countries to provide a second dose of MCV. Critical inputs to the estimation of measles mortality in India are accurate estimates of CFR. According to a review of 25 studies published during 1970–2008 from 12 states, the median case fatality ratio was 1.63% [5]. New estimates are needed from additional states, especially those with low MCV coverage, and in the period after the introduction of a second dose of measles vaccine and the recently-concluded measles catch-up immunization campaign (MCUP) [6].

Bihar, one of the north Indian states, is consistently having lower rates of childhood vaccination. MCV1 coverage was only 58% in 2008 [4], increasing to 76% in 2010–11 according to the Annual Health Survey [7]. MCUP were conducted in the state during December 2010–January 2011 in five districts and during November-December 2011 in another 15 districts, out of the total 38 districts, followed six months later by the introduction of a second dose of MCV into the routine immunization schedule.

Since August 2011, WHO-National Polio Surveillance Project (NPSP) – primarily established for polio surveillance – has been supporting measles surveillance in the state. To the best of our knowledge, no information is available about measles CFR in Bihar. In order to better define measles CFRs in India and specifically in Bihar and identify risk factors associated with mortality, we conducted a retrospective cohort study in areas affected by measles outbreaks.

## Materials and Methods

### Routine Surveillance System for Measles in Bihar

As a part of WHO-NPSP supported measles outbreak surveillance, all reporting units included in Acute Flaccid Paralysis surveillance in the state send weekly reports of suspected measles cases (defined as fever with maculopapular rash with any of the following: cough, coryza or conjunctivitis) to the district [8]. Occurrence of > = 5 cases of suspected measles or any death due to measles in a block (administrative sub-division) in a week is considered as a trigger [8]. In response to the trigger, local health authorities conduct a preliminary investigation to search for additional cases. If preliminary investigations reveal clustering of measles cases (>20) and/or measles deaths, a detailed investigation is conducted with a house-to-house search for suspected measles cases, collection of blood specimens for laboratory confirmation and administration of age appropriate doses of vitamin A to all measles cases identified and appropriate treatment as needed [8]. For laboratory confirmation, five sera samples are collected from suspected measles cases and tested for IgM antibodies against measles using WHO-prequalified ELISA tests; samples negative for measles are tested for rubella. Measles outbreaks are those with > = 2 samples positive for IgM antibodies against measles and <2 samples positive for rubella; rubella outbreaks are those with > = 2 samples positive for IgM antibodies against rubella and <2 samples positive for measles; mixed outbreaks are those with > = 2 samples positive for IgM antibodies against measles and > = 2 samples positive for rubella, and outbreaks of diseases other than measles or rubella are those with <2 samples positive for measles and <2 samples positive for rubella [Ministry of Health and Family Welfare, Government of India. Standard Operating Procedures for Measles Outbreak Investigations, April 2011, unpublished document].

### Sampling Frame and Sampling Procedure

During 1 October 2011 to 30 April 2012, measles surveillance system in Bihar was notified of 136 suspected outbreaks (triggers), 47 of which were further investigated. Based on the laboratory criteria mentioned above, 31 outbreaks were due to measles, 2 were due to rubella and 14 were negative for both measles and rubella. In four of the 31 measles outbreaks, one serum sample each was also positive for rubella. All 31 measles outbreaks occurred in 11 districts that had not yet conducted the MCUP campaign. During this period no outbreaks were reported from the seven other districts that had not yet held the MCUP campaign or from the 20 districts that had already conducted MCUP campaigns during 2010–2011.

For the study, 50% of outbreaks were selected from the 8 districts reporting >1 outbreak using probability proportional to size of the outbreak, and all outbreaks from 3 districts reporting a single outbreak. Each laboratory confirmed outbreak was considered as a cluster. All *tolas* (section of the village)/urban localities surveyed by the district health authorities during the outbreak investigation/response were considered as the geographical boundary of the selected cluster and all households in these *tolas*/urban localities as the cluster size (Table 1). In total, we selected 18 outbreaks in 11 districts out of the total 31 measles outbreaks. Of the 90 sera collected for laboratory confirmation from these 18 outbreaks, 68 were positive for IgM antibodies against measles. The remaining 22 sera were tested for IgM antibodies against rubella, of which 2 samples, one each from 2 outbreaks, were positive.

**Table 1:** Laboratory confirmed measles outbreaks detected during October 2011 and April 2012 and sampling details.

	Laboratory confirmed outbreaks detected by the surveillance system during 1 October 2011–30 April 2012				Laboratory confirmed outbreaks selected for the measles CFR study		
District	Number of outbreaks	Number of measles cases reported	Number of village *tolas* affected	Number of households in the village tolas	Number of outbreaks	Number of village *tolas*	Number of households
Banka	2	82	7	1049	1*	6	927
Darbhanga	9	528	31	5156	5	11	1505
Khagaria	1	39	1	539	1	1	544
Madhepura	1	176	4	278	1	4	280
Madhubani	2	1048	49	3961	1	41	3210
Purnia	2	112	4	425	1	1	92
Saharsa	3	149	10	816	2	7	659
Samastipur	2	96	7	1522	1	5	909
Sitamarhi	1	56	1	125	1	1	125
Supaul	6	546	21	1901	3*	9	646
Vaishali	2	88	2	123	1	1	179
**Total**	**31**	**2920**	**137**	**15895**	**18 (58.1%)**	**87 (63.5%)**	**9076 (57.1%)**

*One serum sample from an outbreak in Banka and another sample from an outbreak in Supaul were positive for IgM antibodies against rubella.

### Data Collection

Data collection occurred during September–October 2012. All the houses in the given cluster were surveyed by study teams comprising of a trained field volunteer/external monitor registered with the district NPSP units in Bihar and a local guide (vaccinator or Accredited Social Health Activist) working in the same area. The study team, using a pre-tested structured questionnaire, conducted a house-to-house survey to identify measles cases and measles deaths occurring between 1 October 2011 and 30 April 2012, a period 5–11 months before data collection. The start and end dates for this recall period coincided with major festivals in the state.

After obtaining written informed consent from the family head, survey teams first enumerated all individuals residing in the given household. The teams then followed a two-step strategy to retrospectively identify measles cases and measles deaths. First, they listed all household members and enquired if anyone experienced fever with rash (locally known as *maata*) during the reference period. If so, the teams interviewed the mothers or the care-takers of such cases, dead or alive and collected detailed information about clinical and treatment details and vaccination history. We also collected information about the type and distribution of the rash. Cases were only considered as measles based on the operational definition given below. Every measles death was verified by NPSP-Surveillance Medical Officer. A local event calendar was prepared and used to determine the date of onset of rash and death.

### Operational Definitions

A resident was any person, dead or alive, who lived in the area of a confirmed measles outbreak for at least 12 months before the interview. A measles case was any resident who met the WHO measles case definition– history of fever and maculopapular rash, and one or more of cough, coryza or conjunctivitis [9]. Operationally, any *maata* case with one or more of cough, coryza and conjunctivitis and having *khasra*-(local term for measles) like rash or rash all over the body was considered as a measles case [10]. A measles associated death was defined as death of a person with measles within 30 days of rash onset, unless the death was from an accident or other unrelated causes [9], [11]. A child was considered to be vaccinated against measles based on the vaccination card or mother’s history if the card was not available. Families having the Below Poverty Line (BPL) card issued by the state authorities on the basis of monthly income were considered as having income below poverty line. Scheduled Castes/Scheduled Tribes (SC/ST) are the caste/tribal groups which are included under articles 341 and 342 of Indian constitution [12]. They are one of the most disadvantaged socio-economic groups in India.

### Data Analysis

Data were double entered and processed using STATA (version 11) software. All analyses, including calculation of CFRs and 95% confidence intervals, were done using sampling weights to account for the probability of selection at the sampling stage and using methods for cluster sampling. We conducted univariate analysis to identify the risk factors for mortality after measles. Variables with P≤0.2 were included in the multiple logistic regression analysis. We used median to dichotomize the interval between occurrence of first case and outbreak response.

### Human Subject Protection

The study protocol was approved by the Institutional Human Ethics Committee of National Institute of Epidemiology, Chennai. Written informed consent was obtained from the heads of all the households surveyed.

## Results

**Figure 1:**
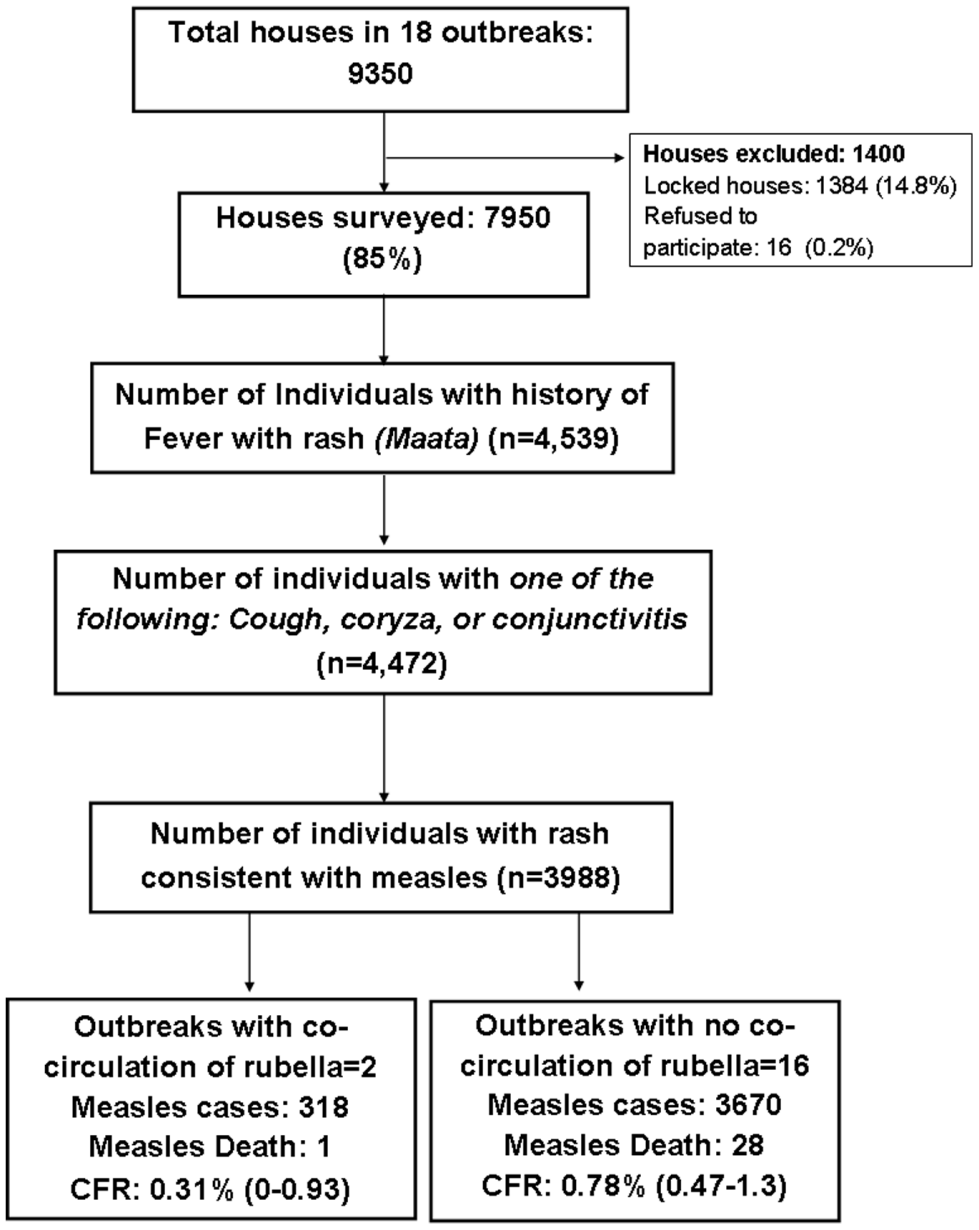
Flowchart showing measles case ascertainment, Bihar, India, 2011–12.

We surveyed 9350 households covering a population of 54,297 from 87 village *tolas/*urban localities from 18 outbreaks in 11 districts. Of these, 1384 (14.8%) households were locked while 16 (0.2%) refused to participate (Fig. 1).

### Measles Cases

In the 18 outbreaks, 4,539 individuals had a history of fever and rash during the reference period. Of these, 4,472 (98.5%) had one or more of cough, coryza or conjunctivitis and 3,988 had rash consistent with maculopapular rash of measles (Fig. 1). In 2 outbreaks, one serum sample each was positive for rubella. As confirmation of rubella could indicate co-circulation of the two viruses, and because not all cases were laboratory confirmed, for the main analysis we included only 16 outbreaks without laboratory-confirmed rubella cases. Tables S2 and S3 in the File S1 show the results including all outbreaks visited. The initial investigation of the 16 outbreaks, conducted by the district health authorities, found 1868 cases overall with a median of 47 cases [inter-quartile range (IQR): 36–86] per outbreak. Through the door to door search a total of 3670 measles cases were found in these outbreaks with a median of 114 (IQR: 80–141) per outbreak.

Measles cases were mainly of Hindu religion (68%), and belonged to families having income below poverty line (76%). Seventy three percent of the cases were of general or other backward caste (G/OBC) while the remaining belonged to SC/ST. Most (55%) cases were > = 5 years of age (median age 5.4 years) and 27% gave history of measles vaccination. Measles vaccination status was different by age group (under-five: 34.4%, > = 5 years: 18.6%, p = 0.0000). About one third of the measles cases had received at least one dose of vitamin A during their illness –1182 received one dose while 106 received two or more doses. Most (n = 2689, 73%) cases reported seeking treatment from traditional healers and 267 (7%) did not seek any treatment. The remaining patients sought care from private (n = 322, 9%) or public health facilities (n = 115, 3%) or purchased medicines from pharmacy (n = 277, 8%).

Socio-economic status differed significantly between the measles cases belonging to SC/ST and G/OBC: majority of the measles cases belonging to SC/ST were BPL (SC/ST: 92.3% vs G/OBC: 70.2%, p = 0.0000), had illiterate family head (SC/ST: 78.1% vs G/OBC: 63.1%, p = 0.03). More cases in SC/ST were also younger (SC/ST: 51.5% <5 years of age vs. G/OBC: 41.8%, p = 0.00001). Although there was no difference between the treatment seeking behaviour by caste and MCV1 coverage by caste, the coverage of vitamin A administration was higher among measles cases belonging to SC/ST (SC/ST: 48.1% vs. G/OBC: 31.2%, p = 0.03).

### Measles Deaths and Case Fatality Rate

Of the 3,670 measles cases in 16 outbreaks surveyed, 28 died within 30 days of rash onset with an overall adjusted CFR of 0.78% (95% CI: 0.47–1.30). Fourteen cases each died during the first and second week of rash onset. As compared to the surveillance system, we identified 14 additional deaths (50% of the total measles deaths); four of whom had died after the investigation of the outbreak. Measles CFR in different districts is presented in Table S1 in File S1.

CFR decreased with age, from 2.3% in infants to 1.1% in children aged 1–4 y to 0.42% in children aged > = 5 y. The CFR was significantly higher cases who did not receive vitamin A during the illness, those who belonged to families whose head was illiterate, had an income below the poverty line or belonged to SC/ST (Table 2). CFR tended to be higher among single cases or primary cases in households compared to secondary cases, though the difference was not statistically significant. Among the under-five as well as older children, CFR was significantly higher among children belonging to SC/ST compared to G/OBC (Table 3). Considering only measles cases who did not receive vitamin A during outbreak response, the adjusted CFR was significantly higher among children belonging to SC/ST as compared to G/OBC (Table 3).

**Table 2:** Measles case fatality rate by selected variables, in 16 outbreaks, Bihar, India, October 2011 to April 2012.

Variable	Measlescases (%)	Measlesassociated death	Adjusted CFR(%) (95% CI)	Odds Ratio(95% CI)	P
All	3670	28	0.78 (0.47–1.30)		
Age (years)					
> = 5	2034 (55.4)	8	0.42 (0.26–0.67)	1	
1–4	1423 (38.8)	15	1.06 (0.52–2.17)	2.57 (1.27–5.18)	0.001
<1	213 (5.8)	5	2.29 (1.04–4.95)	5.60 (2.27–13.79)	0.012
<5	1636 (44.6)	20	1.22 (0.66–2.27)		
Sex					
Male	1876 (51.1)	12	0.67 (0.36–1.24)	1	
Female	1794 (48.9)	16	0.90 (0.54–1.49)	1.36 (0.84–2.19)	0.196
Religion					
Others	1179 (32.1)	6	0.50 (0.30–0.83)	1	
Hindu	2491 (67.9)	22	0.91 (0.48–1.71)	1.83 (0.93–3.59)	0.076
Caste					
General/OBC	2679 (73.0)	11	0.42 (0.20–0.88)	1	
Scheduled caste/Tribe	991 (27.0)	17	1.72 (1.05–2.81)	4.12 (2.37–7.19)	0.000
Education of head of the household					
Primary and above	1190 (32.4)	2	0.21 (0.06–0.68)	1	
Illiterate	2480 (67.6)	26	1.06 (0.60–1.88)	5.23 (1.21–22.61)	0.029
Family income					
Above poverty line	893 (24.3)	3	0.38 (0.12–1.17)	1	
Below poverty line	2777 (75.7)	25	0.91 (0.60–1.37)	2.41 (1.10–5.72)	0.030
Measles vaccination status					
Vaccinated	988 (26.9)	7	0.83 (0.36–1.90)	1	
Not vaccinated/Unknown*	2682 (73.1)	21	0.77 (0.48–1.21)	0.92 (0.50–1.71)	0.783
Vit A given during illness					
Yes	1288 (35.1)	2	0.12 (0.06–0.27)	1	
No/unknown**	2382 (64.9)	26	1.15(0.65–2.03)	9.31 (3.88–22.35)	0.000
Treatment during the illness					
Public/private health facility	437 (11.9)	3	0.55 (0.09–3.28)	1	
Bought from pharmacy	277 (7.5)	1	0.19 (0.02–2.29)	0.35 (0.03–4.61)	0.397
From Traditional healer/No treatment	2956(80.5)	24	0.87 (0.44–1.71)	1.58 (0.15–16.64)	0.683
Treatment at the public health facility					
Yes	115 (3.1)	0	0		
No	3555 (96.9)	28	0.81 (0.48–1.37)		
Median interval in days between onset of first case and notification of outbreak					
<49 days (8 outbreaks)	916 (25.0)	3	0.28 (0.10–0. 79)	1	0.038
> = 49 days (8 outbreaks)	2754 (75.0)	25	0.91 (0.63–1.31)	3.25 (1.08–9.78)	
Median interval between the notification and outbreak response					0.093
<17 days (7 outbreaks)	2698 (73.5)	24	0.89 (0.58–1.34)	1	
> = 17 days (9 outbreaks)	972 (26.5)	4	0.42 (0.19–0.92)	0.47 (0.19–1.15)	
Median interval between the onset and outbreak response					0.280
<70 days (8 outbreaks)	802 (21.9)	4	0.42 (0.11–1.52)	1	
> = 70 days) (8 outbreaks)	2868 (78.1)	24	0.86 (0.56–1.32)	2.05 (0.52–8.08)	
Median number of cases per outbreak					
<114 cases (7 outbreaks)	717 (19.5)	4	0.52 (0.19–1.45)	1	
> = 114 cases (9 outbreaks)	2953 (80.5)	24	0.84 (0.52–1.35)	1.62 (0.52–5.07)	0.378
Order of case within the household					
>1 case in household, not first case	1821 (49.6)	11	0.66 (0.37–1.19)	1	
>1 case in household, first case	1008 (27.5)	11	1.05 (0.68–1.62)	1.59 (0.86–2.96)	0.132
Single case	841 (22.9)	6	0.71 (0.25–1.98)	1.07 (0.54–2.14)	0.832
Order of cases within household					
>1 case in household, not 1^st^ case	1821 (49.6)	11	0.66 (0.37–1.19)	1	
>1 case in household,1^st^ case/single case in household	1849 (50.4)	17	0.90 (0.52–1.54)	1.35 (0.87–2.10)	0.161

*MCV1 status of 455 (12.4%) of children was unknown, **Vitamin A administration status of 179 (4.9%) children was unknown.

**Table 3:** Measles case fatality rate among SC/ST and G/OBC cases stratified by age group and vitamin A administration, Bihar, India.

	Measles death	Measles cases	CFR	95% CI	P
**Age <5 years**					
G/OBC	10	1125	0.90	0.44–1.83	0.004
SC/ST	10	511	1.92	1.01–3.62	
**Age> = 5 years**					
G/OBC	1	1554	0.07	0.03–0.19	0.000
SC/ST	7	480	1.51	0.86–2.64	
**Vitamin A given**					
G/OBC	2	817	0.20	0.10–0.41	0.071
SC/ST	0	471	0	0	
**Vitamin A not given**					
G/OBC	9	1862	0.53	0.21–1.28	0.000
SC/ST	17	520	3.32	2.21–4.95	

The median interval between the occurrence of the first case in these outbreaks and the notification of the outbreak was 49 days (IQR: 20–67 days), the interval between notification and outbreak response was 17 days (IQR: 9–24 days), and the median interval between the onset of first case and initiation of outbreak response was 70 days (IQR = 39–82 days). Measles cases occurring after the outbreak investigation had significantly lower coverage of vitamin A as compared to those occurring before (31.7% vs 38.7%, p = 0.01).

On multivariate analysis, independent risk factors associated with higher odds of death included age <5 years, SC/ST status, and non-administration of vitamin A. Outbreaks with longer interval between the rash onset of first case and the notification of outbreak also had higher deaths (Table 4). Though the CFR was higher among measles cases belonging to SC/ST and who did not receive vitamin A, a zero-cell value in table 3 (zero deaths among the SC/ST cases who received vitamin A) prevented the estimation of odds ratio for the interaction term for caste and vitamin A treatment.

**Table 4:** Multiple logistic regression analysis of the risk factors associated with measles death during 16 outbreaks in Bihar, India, October 2011 to April 2012.

Variables	Adjusted odds ratio	95% CI		P
Age (year)				
<1	4.95	2.05	11.94	0.002
1–4	2.73	1.20	6.21	0.020
> = 5	1			
Female sex	1.40	0.84	2.30	0.178
Hindu religion	0.78	0.26	2.33	0.642
Income below poverty line	1.22	0.55	2.75	0.601
Illiterate head of the household	4.45	0.80	24.77	0.084
Scheduled caste/tribe	4.86	1.72	13.75	0.005
Vitamin A not given during illness	11.65	4.53	29.97	0.000
First cases in the household vs second or higher cases in household	1.45	0.93	2.28	0.098
Interval between the rash onset of first case and notification of outbreak (> = Median)	3.86	1.27	11.72	0.021
Interval between notification of outbreak and outbreak response (> = Median)	1.31	0.44	3.93	0.603

## Discussion

This community-based study of measles deaths in 16 outbreaks in Bihar, India found that the overall CFR was 0.78%, but varied from 2.3% in children less than one year to 1.1% in children 1–4 years of age to 0.4% in children five years and older. Measles mortality was also higher among children who belonged to SC/ST, those families whose heads were illiterate, and among children who were the first or only case in a household. Most deaths occurred among measles cases who did not receive vitamin A. The CFR observed in our study was lower than the median CFR of 1.63% reported in a review based on 25 studies done earlier in India [5]. Comparisons with earlier studies is difficult as 8 used prospective case ascertainment, all in the pre-vaccination era, and though the remaining studies used house-to-house surveys for cases, most surveys were done right at the end of an outbreak or in periods of “endemic disease”, with some cases likely to be surveyed before 1 month had elapsed since rash onset. Most previous surveys in India did not clearly describe use of household lists and lists of cases in order to maximize the ascertainment of deaths. The CFR in Bihar was comparable to that found in Nepal, where the CFR was 1.1% (95% CI: 0.5–2.3) overall, 3.5% in cases <1 year of age, 2% in cases 1–4 years, and 0.2 in cases 5–14 years of age [2]. Methods for ascertainment of measles cases and death in the current study were similar to the study conducted in Nepal. The findings of our survey have implications in further reducing the measles CFR in Bihar as well as in India. CFR estimates will also improve the estimates of measles deaths in India.

During 2000–2011, the global estimates of measles mortality decreased by more than three-quarters, however in the south-east Asian region the decrease was only by half, with India alone accounting for 36% of the total deaths in 2011 [13], [14]. The measles mortality model developed by the World Health Organization uses vaccination coverage estimates and surveillance data to estimate the number of measles cases by country. The estimated age distribution of cases and case fatality rates are then used to calculate the number of deaths [13], [15]. The findings of estimated number of measles deaths from India should change with the completion in 2013 of the MCUP in 14 states with MCV1 coverage <80% and introduction of a second dose of measles vaccine in routine in all states. Having updated estimates of measles CFR in high-burden countries such as India will be critical for accurately estimating national, regional and global mortality.

In Bihar, by April 2012, no outbreaks had been detected by the surveillance system in the 20 districts of Bihar where MCUP had been conducted during 2010 and 2011 (Measles surveillance in Bihar, unpublished data) indicating a potential early impact of the MCUP. The coverage of MCUP in Bihar using administrative data was 93% (NPSP, unpublished data). A significant decline in the number of measles following MCUP was also reported in the states of Maharashtra, Rajasthan and Gujarat (NPSP, unpublished data). These states reported 3188 measles cases one year before the MCUP compared to 69 cases reported within one year of MCUP (NPSP, unpublished data). In the 11 study districts of Bihar where MCUP had not been conducted by 2012, implementation of enhanced measles surveillance with support from WHO India-NPSP probably has still improved the CFR through the detection and laboratory confirmation of suspected outbreaks as well as their investigation and appropriate response including administration of vitamin A. MCUP in the remaining 18 districts of Bihar was conducted during Jan 2013.

The lower overall CFR observed in Bihar might be related to the older age of measles cases. Though CFR was higher among under-five children, over half of the measles cases were aged > = 5 years and the CFR was lowest (0.41%) in this age group. The declining CFR with age observed in our study is consistent with the studies conducted in other countries as well as community based studies in India [5], [11]. The higher proportion of cases among older children reflects the improvement in MCV1 coverage. MCV1 coverage in Bihar has increased from 40.4% during 2005–6 [16], to 76% during 2010–2011 [7].

The association with vitamin A and lower measles mortality found in Bihar is consistent with reports from several studies conducted in other countries [1], [17]. The association between measles mortality and vitamin A was modified by caste, with higher CFR among those not receiving vitamin A in SC/ST. SC/ST is one of the most disadvantaged socio-economic groups recognized by the Constitution of India. In Bihar, there were significant differences between the measles cases belonging to SC/ST and G/OBC with respect to their socio-economic status as well as age distribution. These factors could partly explain the differences in CFR. It is therefore necessary to improve coverage with two doses of measles vaccine in these communities and during measles outbreaks prioritize them for vaccination activities and for vitamin A administration.

Our survey identified nearly twice the number of measles cases as compared to the cases identified by the surveillance system, with a reporting fraction of 51%. Overall, <5% measles cases received treatment from public health facilities which is likely to affect the sensitivity of the surveillance system and may be the reason for delayed notification of outbreaks [18]. The majority of measles cases in these outbreaks sought treatment from traditional healers. Similar health seeking behaviour was also reported by other studies in India [19], [20]. In Bihar, the interval between the occurrence of the first case and the notification of outbreak was long, with investigation and response beginning after a median interval of two weeks.

Late notification of outbreaks resulted in a lower overall coverage of vitamin A administration to measles cases and thereby higher case fatality. Our survey detected 50% more measles deaths than the surveillance system and 29% (4/14) of these cases had died after the investigation of the outbreak. Measles cases occurring after the investigation of the outbreak had lower coverage of vitamin A. These findings suggest poor adherence to vitamin A treatment of measles cases occurring after the investigation of outbreak.

Several studies have reported milder infection, reduced complications and lower CFR among measles vaccinated children [11], [21], [22]. It has been suggested that higher measles vaccination coverage lower mortality rates directly through reduced disease incidence and indirectly by increasing the median age at infection, resulting in more cases in older age groups with lower mortality rates, or by decreasing incidence, resulting in a lower intensity of transmission [11]. These studies often relied on records of vaccination status before the outbreak [21] or had higher proportions of cases vaccinated [2], [23]. No difference in CFR between vaccinated and unvaccinated children was found in our study, with only 27% of cases with a history of vaccination. Assuming 85% efficacy of the single dose of measles vaccine, 27% measles cases with history of vaccination translates to MCV1 coverage of 71% in outbreak areas [24], which was comparable to the MCV1 coverage of 78% reported in the Annual Health survey – 2010–11 (7). In settings such as Bihar, with low retention of immunization cards (15% vaccinated cases had immunization card in our survey), multiple round of vaccinations, and high proportion of illiterate families, collecting reliable information about vaccination, especially from measles cases who died remains a challenge.

Our study was subject to at least five limitations. First, though we selected laboratory confirmed outbreaks for the survey, due to the retrospective nature of the study, it is possible that some measles cases might have been misclassified, as the case status was based on the symptoms reported by the parents/care-takers. Parents might have inaccurately recalled the occurrence of measles cases in the family as well as some risk factor information. In particular, parents of children dying of measles are more likely to report the risk factor information in a socially desirable direction in order to avoid criticism by the interviewer or out of guilt. We tried to reduce the recall bias by limiting the recall period for measles cases included in the survey to be the period 5–11 months before data collection, and using a local event calendar to aid parents recall. We also followed recommendations to first enumerate all the household members at the beginning of the recall period and use this list to identify who developed rash illness and died within 30 days of rash onset [10]. Interviewing mothers to collect information about the child’s illness and focusing the study on laboratory-confirmed outbreaks increases the positive predictive value of the clinical diagnosis of measles [10]. We used local terms and standardized clinical algorithm to ascertain the cases and deaths. Second, about 15% of the households were locked at the time of the visit and could not be surveyed. We collected information about measles deaths from the village head and the local village guide prior to the survey. No death was reported from the households that were locked. However, as the information about measles cases was not collected from these houses, we might have overestimated the CFR. Third, measles surveillance in the country is presently geared to capture larger outbreaks with at least 20 cases. Measles cases and deaths occurring in smaller outbreaks were not included in our sampling frame. Fourth, the confirmation of measles outbreaks is based on laboratory testing of only five sera samples. Although we excluded 2 outbreaks with co-circulation of rubella, rubella may have been co-circulating with measles in the outbreaks included in the analysis. However, requiring laboratory confirmation of all cases would be difficult to ensure and ethically challenging while at the same time ensuring complete case ascertainment and information on outcomes 1 month after rash onset [10].Lastly, the study results might not apply to other time periods or other Indian states as measles incidence and severity varies by season, epidemic cycle, geography and levels of population immunity to measles.

This case fatality rate survey followed expert guidelines in the choice of a retrospective survey to ascertain all cases and deaths due to measles in the affected areas of Bihar. The inclusion of only outbreaks with at least 20 cases provided a sampling frame of confirmed outbreaks but may have introduced some bias as smaller outbreaks would necessarily have been excluded. Any smaller outbreaks that occurred in Bihar by definition would have involved many fewer cases relative to the ones chosen and probably no deaths. Their exclusion should not have significantly biased the CFR estimates. Given the status of measles control in Bihar it is likely that small outbreaks would grow and coalesce with neighbouring outbreaks to reach a size where they would have been detected, investigated, and eligible for inclusion in this study.

In conclusion, the overall measles CFR in Bihar was found to be lower than other studies from India. The lower rate may represent fewer cases in these outbreaks in groups where CFR was higher, such as young children. Children belonging to SC/ST groups however still have high CFR. Delayed notification of outbreaks might also have led to higher mortality. More systematic communication efforts are needed to promote parents to ensure their children receive two doses of measles vaccine and bring children with measles to public health facilities for treatment of measles complications. Improvements in vaccination coverage should reduce the size and number of future outbreaks in Bihar and thereby reduce the number of measles deaths. Further reductions in measles case fatality could result from timely outbreak investigation and focusing vitamin A administration to SC/ST communities and to children <5 years of age. Although the study was based in 11 districts of Bihar, the recommendations for reducing measles case fatality are applicable to measles outbreaks response in other Indian states. Finally, similar surveys need to be conducted in other states and repeated periodically in order to generate reliable estimates of measles mortality and monitor progress in measles control in India.

## Supporting Information

File S1 Table S1, Selected characteristics of 16 measles outbreaks by district, Bihar, 2011–12. Table S2, Measles case fatality rate by selected variables in 18 outbreaks, Bihar, India, October 2011 to April 2012. Table S3, Multiple logistic regression analysis of the risk factors associated with measles death during 18 outbreaks in Bihar, India, October 2011 to April 2012.
(DOCX)

## References

[1] Stein CE, Birmingham M, Kurian M, Duclos P, Strebel P (2003) The global burden of measles in the year 2000: a model that uses country-specific indicators. J Infect Dis 187 Suppl 1S8–14. 12721886 10.1086/368114

[2] Joshi A, Luman E, Nandy R, Subedi B, Liyanage J, Wierzba T (2009) Measles deaths in Nepal: estimating the national case-fatality ratio. Bull World Health Organ 87: 456–465. 19565124 10.2471/BLT.07.050427 PMC2686206

[3] Wolfson LJ, Strebel PM, Gacic-Dobo M, Hoekstra EJ, McFarland JW, et al (2007) Has the 2005 measles mortality reduction goal been achieved? A natural history modelling study. Lancet 369: 191–200. 17240285 10.1016/S0140-6736(07)60107-X

[4] UNICEF. Coverage evaluation survey 2009. Available: http://www.unfpa.org/sowmy/resources/docs/library/R309_UNICEF_2010_INDIA_2009CoverageSurvey.pdf. Accessed on Sep 2013.

[5] Sudfeld CR, Halsey NA (2009) Measles case fatality ratio in India: a review of community based studies. Indian Pediatr 46: 983–989. 19955580

[6] Ministry of Health and Family Welfare, Govt of India (2010) Measles Catch-up Immunization Campaign - Guidelines for Planning and Implementation 2010. Available: http://www.unicef.org/india/Operational_guideline_for_Measles_catch_up_immunization_campaign__MoHFW_GoI_(English).pdf. Accessed on 26 Jan 2014.

[7] Govt of India. Annual Health Survey 2010–2011. Available: http://www.censusindia.gov.in/vital_statistics/AHSBulletins/AHS_Baseline_Factsheets/Bihar.pdf. Accessed on 10 Sep 2013.

[8] Ministry of Health and Family Welfare, Government of India. Measles surveillance and outbreak investigation. Field Guide. 2005. Available: http://www.npspindia.org/download/Measles%20Guide.pdf. Accessed on 10 Sep 2013.

[9] WHO (1993) Generic protocol for determining measles case fatality rates in a community, either during an epidemic or in highly endemic areas [WHO document WHO/EPI/GEN/93.03]. Geneva.

[10] Cairns KL, Nandy R, Grais RF (2010) Challenges in measuring measles case fatality ratios in settings without vital registration. Emerg Themes Epidemiol 2010 7: 4. 10.1186/1742-7622-7-4 PMC2918600 20642812

[11] Wolfson LJ, Grais RF, Luquero FJ, Birmingham ME, Strebel PM (2009) Estimates of measles case fatality ratios: a comprehensive review of community-based studies. Int J Epidemiol 38: 192–205. 19188207 10.1093/ije/dyn224

[12] Ministry of Social Justice and Empowerment, Govt of India. Report of Advisory Committee on the Revision of Lists of Scheduled Castes and Scheduled Tribes, 1965 Available: http://www.socialjustice.nic.in/pdf/ac-listofscst1965.pdf. Accessed on 4 Feb 2014.

[13] Simons E, Ferrari M, Fricks J, Wannemuehler K, Anand A, et al (2012) Assessment of the 2010 global measles mortality reduction goal: results from a model of surveillance data. Lancet 379: 2173–8. 22534001 10.1016/S0140-6736(12)60522-4

[14] Centers for Disease Control and Prevention (2013) Global Control and Regional Elimination of Measles, 2000–2011 MMWR Morb Mortal Wkly Rep. 62: 27–31. PMC4604839 23325353

[15] Chen S, Fricks J, Ferrari MJ (2012) Tracking measles infection through non-linear state space models. J R Stat Soc Ser C Appl Stat 61: 117–24.

[16] International Institute for Population Sciences (IIPS) and Macro International. National Family Health Survey (NFHS-3), 2005–06: India. Vol 1. Mumbai, India: IIPS; 2007. p.227–33.

[17] D’Souza RM, D’Souza R (2002) Vitamin A for treating measles in children. Cochrane Database Syst Rev 1: CD001479. 10.1002/14651858.CD001479 11869601

[18] Murhekar MV, Roy D, Das PK, Bose AS, Ramakrishnan R, et al (2011) Measles in rural West Bengal, India, 2005–6: low recourse to the public sector limits the use of vitamin A and the sensitivity of surveillance. J Infect Dis. 204 Suppl 1S427–32. 10.1093/infdis/jir062 21666195

[19] Manchanda KS, Kumar V, Bhatnagar V (1980) Understanding of diseases and treatment-seeking pattern of childhood illnesses in rural Haryana, India. Trop Geogr Med 32: 70–6. 7394896

[20] Singh MB (1994) Maternal beliefs and practices regarding the diet and use of herbal medicines during measles and diarrhea in rural areas. Indian Pediatr 31: 340–3. 7896375

[21] Samb B, Aaby P, Whittle H, Seck AM, Simondon F (1997) Decline in measles case fatality ratio after the introduction of measles immunization in rural Senegal. Am J Epidemiol 145: 51–7. 8982022 10.1093/oxfordjournals.aje.a009031

[22] Aaby P, Bukh J, Leerhøy J, Lisse IM, Mordhorst CH (1986) Vaccinated children get milder measles infection: a community study from Guinea-Bissau. J Infect Dis 154: 858–63. 3772165 10.1093/infdis/154.5.858

[23] Coronado F, Musa N, El Tayeb el SA, Haithami S, Dabbagh A (2006) Retrospective measles outbreak investigation: Sudan, 2004. J Trop Pediatr 52: 329–34. 16735363 10.1093/tropej/fml026

[24] Orenstein WA, Bernier RH, Hinman AR (1988) Assessing vaccine efficacy in the field. Further observations. Epidemiol Rev 10: 212–41. 3066628 10.1093/oxfordjournals.epirev.a036023

