# Supplementary material for: Measles Case Fatality Rate in Bihar, India, 2011–12
Source: PLoS One. 2014 May 13;9(5):e96668. doi: 10.1371/journal.pone.0096668 (PMC4019661; doi:10.1371/journal.pone.0096668)
Supplement: File S1 — Table S1, Selected characteristics of 16 measles outbreaks by district, Bihar, 2011–12. Table S2, Measles case fatality rate by selected variables in 18 outbreaks, Bihar, India, October 2011 to April 2012. Table S3, Multiple logistic regression analysis of the risk factors associated with measles death during 18 outbreaks in Bihar, India, October 2011 to April 2012. (DOCX) [file pone.0096668.s001.docx]

**Supplementary Tables**

Table S1: Selected characteristics of 16 measles outbreaks by district, Bihar, 2011-12

| District | Measles cases | % under-five cases | % cases vaccinated against MCV | % cases received vitamin A treatment | % cases belonging to SC/ST | Measles death | CFR (%) |
| --- | --- | --- | --- | --- | --- | --- | --- |
| Darbhanga | 694 | 43.4 | 29.8 | 33.6 | 19.9 | 1 | 0.14 |
| Khagaria | 175 | 30.3 | 73.7 | 10.9 | 8.0 | 0 | 0 |
| Madhepura | 102 | 50.0 | 41.2 | 18.6 | 4.9 | 0 | 0 |
| Madhubani | 1898 | 45.6 | 17.4 | 38.4 | 26.2 | 20 | 1.05 |
| Purnia | 54 | 37.0 | 29.6 | 72.2 | 96.3 | 1 | 1.85 |
| Saharsa | 237 | 41.4 | 20.3 | 16.9 | 43.5 | 4 | 1.69 |
| Samastipur | 130 | 48.5 | 60.8 | 30.8 | 26.2 | 0 | 0 |
| Sitamarhi | 129 | 38.8 | 26.4 | 32.6 | 0.8 | 1 | 0.78 |
| Supaul | 137 | 48.9 | 50.4 | 19.0 | 23.4 | 1 | 0.73 |
| Vaishali | 114 | 59.6 | 29.8 | 88.6 | 100.0 | 0 | 0 |
| **Overall** | **3670** | **44.6** | **26.9** | **35.1** | **27.0** | **28** | **0.78** |

Table S2: Measles case fatality rate by selected variables in 18 outbreaks, Bihar, India, October 2011 to April 2012

| **Variable** | | **Measles cases (%)** | **Measles associated death** | **Adjusted CFR (%)**  **(95% CI)** | **Odds Ratio (95% CI)** | **P** |
| --- | --- | --- | --- | --- | --- | --- |
| **All** | | **3988** | **29** | **0.74 (0.42-1.30)** |  |  |
| Age (years) | |  |  |  |  |  |
|  | >=5 | 2216 (55.6) | 8 | 0.37 (0.21-0.64) | 1 |  |
|  | 1-4 | 1538 (38.6) | 16 | 1.06 (0.54-2.09) | 2.88 (1.63-5.09) | 0.001 |
|  | <1 | 234 (5.9) | 5 | 2.08 (0.91-4.68) | 5.71 (2.40-13.60) | 0.001 |
|  | <5 | 1772 (44.4) | 21 | 1.20 (0.64-2.22) |  |  |
| Sex | |  |  |  |  |  |
|  | Male | 2028 (50.9) | 12 | 0.62 (0.31-1.24) | 1 | 0.159 |
|  | Female | 1960 (49.1) | 17 | 0.87 (0.51-1.48) | 1.40 (0.86-2.29) |  |
| Religion | |  |  |  |  |  |
|  | Others | 1214 (30.4) | 6 | 0.48 (0.29-0.81) | 1 |  |
|  | Hindu | 2774 (69.6) | 23 | 0.85 (0.43-1.69) | 1.77 (0.91-3.44) | 0.090 |
| Caste | |  |  |  |  |  |
|  | General/OBC | 2897 (72.6) | 11 | 0.39 (0.17-0.89) | 1 |  |
|  | Scheduled caste/Tribe | 1091 (27.4) | 18 | 1.64 (0.99-2.69) | 4.23 (2.47-7.27) | 0.000 |
| Education of head of the household | |  |  |  |  |  |
|  | Primary and above | 1327 (33.3) | 2 | 0.16 (0.06-0.48) | 1 |  |
|  | Illiterate | 2661 (66.7) | 27 | 1.03 (0.58-1.84) | 6.36 (1.85-21.86) | 0.006 |
| Family income | |  |  |  |  |  |
|  | Above poverty line | 1006 (25.2) | 3 | 0.33 (0.10-1.16) | 1 | 0.031 |
|  | Below poverty line | 2982 (74.8) | 26 | 0.88 (0.56-1.36) | 2.63 (1.11-6.25) |  |
| Measles vaccination status | |  |  |  |  |  |
|  | Vaccinated | 1160 (29.1) | 8 | 0.78 (0.33-1.82) | 1 |  |
|  | Not vaccinated/Unknown | 2828 (70.9) | 21 | 0.73 (0.44-1.21) | 1.07 (0.58-1.96) | 0.817 |
| Vit A given during illness | |  |  |  |  |  |
|  | Yes | 1411 (35.4) | 2 | 0.11 (0.05-0.25) | 1 |  |
|  | No | 2577 (64.6) | 27 | 1.1 (0.59-2.03) | 9.76 (4.21-22.61) | 0.000 |
| Treatment during the illness | |  |  |  |  |  |
|  | Public/private health facility | 501 (12.6) | 3 | 0.48 (0.09-2.54) | 1 |  |
|  | Bought from pharmacy | 291 (7.3) | 1 | 0.19 (0.02-2.11) | 0.38 (0.03-5.03) | 0.602 |
|  | From Traditional healer/No treatment | 3196 (80.1) | 25 | 0.83 (0.41-1.69) | 1.74 (0.19-15.90) | 0.471 |
| Treatment at the public health facility | |  |  |  |  |  |
|  | Yes | 137 (3.4) | 0 | 0 |  |  |
|  | No | 3851 (96.6) | 29 | 0.77 (0.43-1.37) |  |  |
| Median interval in days between onset of first case and notification of outbreak | | | | |  |  |
|  | <49 days (10 outbreaks) | 1174 (29.4) | 3 | 0.21 (0.06-0.68) | 1 | 0.021 |
|  | >= 49 days (8 outbreaks) | 2814 (70.6) | 26 | 0.92 (0.66-1.29) | 4.53 (1.30-15.79) |  |
| Median interval between the notification and outbreak response | | | | |  | 0.284 |
|  | <16 days (11 outbreaks) | 2956 (74.1) | 24 | 0.81 (0.46-1.40) | 1 |  |
|  | >= 16 days (7 outbreaks) | 1032 (25.9) | 5 | 0.50 (0.25-1.02) | 0.62 (0.25-1.54) |  |
| Median interval between the onset and outbreak response | | | |  |  | 0.140 |
|  | <70 days (10 outbreaks) | 1060 (26.6) | 4 | 0.30 (0.07-1.23) | 1 |  |
|  | >= 70 days) (8 outbreaks) | 2928 (73.4) | 25 | 0.88 (0.59-1.30) | 2.98 (0.67-13.18) |  |
| Median number of cases per outbreak | |  |  |  |  |  |
|  | <114 cases (8 outbreaks) | 777 (19.5) | 5 | 0.61 (0.26-1.47) | 1 |  |
|  | >= 114 cases (10 outbreaks) | 3211 (80.5) | 24 | 0.77 (0.42-1.40) | 1.25 (0.43-3.65) | 0.661 |
| Order of case within the household | |  |  |  |  |  |
| >1 case in household, not first case | | 1964 (49.2) | 11 | 0.60 (0.30-1.2) | 1 |  |
| >1 case in household, first case | | 1090 (27.4) | 12 | 1.08 (0.69-1.67) | 1.80 (0.88-3.68) | 0.102 |
| Single case | | 934 (23.4) | 6 | 0.65 (0.21-1.95) | 1.07 (0.59-1.98) | 0.800 |
| Order of cases within household | |  |  |  |  |  |
| >1 case in household, not 1^st^ case | | 1964 (49.2) | 11 | 0.60 (0.30-1.20) | 1 |  |
| >1 case in household, 1^st^ case/single case in household | | 2024 (50.8) | 18 | 0.88 (0.51-1.52) | 1.46 (0.95-2.26) | 0.081 |

Table S3: Multiple logistic regression analysis of the risk factors associated with measles death during 18 outbreaks in Bihar, India, October 2011 to April 2012

| Variables | | Adjusted odds ratio | 95% CI | | P |
| --- | --- | --- | --- | --- | --- |
| Age (year) | |  |  |  |  |
|  | <1 | 5.00 | 2.06 | 12.11 | 0.001 |
|  | 1-4 | 3.21 | 1.70 | 6.07 | 0.001 |
|  | >=5 | 1 |  |  |  |
| Female sex | | 1.52 | 0.86 | 2.68 | 0.135 |
| Hindu religion | | 0.89 | 0.45 | 1.78 | 0.728 |
| Income below poverty line | | 1.25 | 0.53 | 2.95 | 0.586 |
| Illiterate head of the household | | 5.56 | 1.35 | 22.93 | 0.021 |
| Scheduled caste/tribe | | 5.23 | 2.30 | 11.92 | 0.001 |
| Vitamin A not given during illness | | 14.23 | 6.12 | 33.03 | 0.000 |
| First cases in the household vs second or higher cases in household | | 1.65 | 1.04 | 2.61 | 0.035 |
| Interval between the rash onset of first case and notification of outbreak (>=Median) | | 2.36 | 0.94 | 5.97 | 0.067 |
| Interval between rash onset of first case and outbreak response (>=Median) | | 3.03 | 1.33 | 6.95 | 0.011 |
